# S-Denitrosylation: A Crosstalk between Glutathione and Redoxin Systems

**DOI:** 10.3390/antiox11101921

**Published:** 2022-09-28

**Authors:** Surupa Chakraborty, Esha Sircar, Camelia Bhattacharyya, Ankita Choudhuri, Akansha Mishra, Sreejita Dutta, Sneha Bhatta, Kumar Sachin, Rajib Sengupta

**Affiliations:** 1 Amity Institute of Biotechnology Kolkata, Amity University Kolkata, Action Area II, Rajarhat, Newtown, Kolkata 700135, West Bengal, India; 2 Department of Biological Sciences and Bioengineering, Indian Institute of Technology, Roorkee 247667, Uttarakhand, India; 3 Department of Biosciences, Swami Rama Himalayan University, Jolly Grant, Dehradun 248016, Uttarakhand, India

**Keywords:** antioxidant systems, glutaredoxin, glutathione, glutathione peroxidase, glutathione reductase, glutathione transferase, glutathionylation, nitric oxide, nitric oxide synthases, nitro-oxidative stress, oxidoreductases, peroxiredoxin, thioredoxin, thioredoxin reductase, thioredoxin interacting protein, thioredoxin-related protein 14, reactive oxygen species, reactive nitrogen species, redox homeostasis, redundancy, S-(de)nitrosylation, S-nitrosoglutathione, S-nitrosoproteins, thiol disulfides

## Abstract

S-nitrosylation of proteins occurs as a consequence of the derivatization of cysteine thiols with nitric oxide (NO) and is often associated with diseases and protein malfunction. Aberrant S-nitrosylation, in addition to other genetic and epigenetic factors, has gained rapid importance as a prime cause of various metabolic, respiratory, and cardiac disorders, with a major emphasis on cancer and neurodegeneration. The S-nitrosoproteome, a term used to collectively refer to the diverse and dynamic repertoire of S-nitrosylated proteins, is relatively less explored in the field of redox biochemistry, in contrast to other covalently modified versions of the same set of proteins. Advancing research is gradually unveiling the enormous clinical importance of S-nitrosylation in the etiology of diseases and is opening up new avenues of prompt diagnosis that harness this phenomenon. Ever since the discovery of the two robust and highly conserved S-nitrosoglutathione reductase and thioredoxin systems as candidate denitrosylases, years of rampant speculation centered around the identification of specific substrates and other candidate denitrosylases, subcellular localization of both substrates and denitrosylases, the position of susceptible thiols, mechanisms of S-denitrosylation under basal and stimulus-dependent conditions, impact on protein conformation and function, and extrapolating these findings towards the understanding of diseases, aging and the development of novel therapeutic strategies. However, newer insights in the ever-expanding field of redox biology reveal distinct gaps in exploring the crucial crosstalk between the redoxins/major denitrosylase systems. Clarifying the importance of the functional overlap of the glutaredoxin, glutathione, and thioredoxin systems and examining their complementary functions as denitrosylases and antioxidant enzymatic defense systems are essential prerequisites for devising a rationale that could aid in predicting the extent of cell survival under high oxidative/nitrosative stress while taking into account the existence of the alternative and compensatory regulatory mechanisms. This review thus attempts to highlight major gaps in our understanding of the robust cellular redox regulation system, which is upheld by the concerted efforts of various denitrosylases and antioxidants.

## 1. Introduction

Nitric oxide (NO), a tiny, lipophilic, redox-active molecule, acts as a catalyst for the activation of the effector Guanylyl cyclase, which in turn mediates the creation of cyclic-GMP (cGMP) from GTP, triggering an intracellular chain of signaling events when present at the right concentration (low nanomolar levels). The relevance of the molecule in redox signaling and homeostasis is exemplified by intracellular amplification of the message conveyed by NO to the cell and its subsequent translation into large-scale alterations seen in both the extracellular and intracellular milieu. Nitric Oxide Synthases (NOSs) are enzymes that produce NO; there are three different isoforms of NOS in mammals, including nNOS (neuronal NOS), found in cardiomyocytes, gastrointestinal smooth muscles, neurons, skeletal muscles, etc., iNOS (inducible NOS), present predominantly in cells of the immune system, such as macrophages, lung fibroblasts, mast cells, Kupfer cells and neutrophils, and eNOS (endothelial NOS), in the endothelial cells and epithelial cells of the mucosal lining. NO functions as a neurotransmitter, smooth muscle relaxant, vasodilator, and, reportedly, also as an antibacterial and anti-tumor agent under normal physiological circumstances. However, disruptions in the regulatory systems that keep NO levels in check could result in an excess of NO and subsequently other free radicals, which could cause a variety of illnesses such as carcinomas, ulcerative colitis, multiple sclerosis, and juvenile diabetes, in addition to direct tissue toxicity and vascular collapse [1,2,3,4].

Free radicals are extremely reactive compounds with valence electrons that are unpaired and have a short half-life. They are produced inside the cell as intermediates of several redox reactions and are crucial for redox signaling in the body. Increased intracellular free radical content can be caused by unhealthy diets lacking important antioxidants, ionizing radiation and UV radiation, pollution, and direct exposure to harmful chemicals such as paraquat. Free radicals can be generically categorized into reactive oxygen species (ROS) and reactive nitrogen species (RNS). ROS comprises toxic intermediates of oxygen metabolism, such as superoxide (O_2_^−^), hydroxyl (HO.), alkoxyl, peroxyl, and hydroperoxyl radicals, along with hydrogen peroxide (H_2_O_2_). Apart from being generated as metabolic by-products in the mitochondria by complex I and complex III, ROS production has also been directly associated with the endoplasmic reticulum, peroxisomes, and NADPH Oxidase (NOX), in addition to metal-induced ROS production in Fenton-like reactions. On the other hand, as the name implies, RNS comprises reactive nitrogen intermediates, such as NO, nitrogen dioxide (NO_2_**.**), and peroxynitrite (ONOO^-^) [5,6]. Owing to their potential for damage, immune cells such as neutrophils and macrophages use ROS and RNS as weapons against infectious agents; the drastic rise in iNOS activity during bacterial, viral, and metastatic invasions could be cited as evidence. Another important function of NO is post-translational control of protein function through covalent modification of amino acids such ascysteine thiol(ates)s, which results in the formation of S-nitrosylated proteins/S-nitrosoproteins (PSNOs), which may either have positive physiological effects (such as the role of S-nitrosoglutathione (GSNO) as an endogenous bronchodilator and NO-mediated activation of platelet specific integrin αIIbβ3) or negative pathological outcomes (as is the case in neurodegenerative diseases, and various respiratory, metabolic, and cardiac ailments) [7,8,9,10,11].

One can gauge the clinical impact of free radical overload and improper oxidative modifications of proteins by examining the myriad of molecular malfunctions and signal misfiring caused by excessive ROS/RNS, which ultimately result in cell death or neoplasia due to the inactivation or hyperactivation of various kinases/phosphatases such as the phosphatase and tensin homolog (PTEN). A number of neurodegenerative and protein aggregation disorders, including frontal temporal dementia (FTD), amyotrophic lateral sclerosis (ALS), Alzheimer’s, Parkinson’s, and Huntington’s diseases, have been linked to disruptions in mitochondrial homeostasis, which is consistent with the fact that mitochondria are the primary intrinsic source of ROS production, and hence are unquestionably the most vulnerable to their own output [7,8].

A daily supply of antioxidants, either from food or other nutraceuticals, is required to counteract exposure to exogenous and endogenous prooxidants or chemicals that induce a burst of ROS/RNS within the body, particularly because the levels of beneficial antioxidants decline with age. Antioxidants are chemicals that control the levels of ROS and RNS within cells and, in some situations, undo abnormal oxidative alterations to biomolecules. Flavonoids such as quercetin, luteolin, ascorbic acid, and α-tocopherol are notable antioxidants that can be found in abundance in ginseng, withaferin, ginkgo extracts, green tea, and red wine. However, one must be careful, as consuming antioxidants in excess of a specific amount may also be dangerous because it may result in reductive stress, a phenomenon that has only recently been identified. Hence, in addition to controlling the number of free radicals in the cell, antioxidants also make sure that other antioxidants and de-nitrosylases, such as reduced glutathione (GSH), thioredoxin (Trx), and glutaredoxin (Grx), do not suffer from a deficiency [12,13,14,15]. De-nitrosylases are a collection of small molecules and enzymatic systems that are deployed by the cell to reverse the harmful, aberrant modifications of cysteine thiols of key intracellular proteins. The existence of an ornately designed network of multiple denitrosylases indicates the presence of a combinatorial approach towards denitrosylation, facilitated by the synergistic cum complementary, and additive actions of the above-mentioned (and other) denitrosylating systems, ascribable to their ‘redundant’ and ‘substrate specific’ nature, respectively. The current article aims to elucidate some key features (such as ‘redundancy’ and ‘substrate selectivity’) of some of the well-known denitrosylating systems, thereby attempting to bring forth the remedial potential of such agents and opening up a hitherto unexplored therapeutic perspective. GSH is the most significant of these systems, followed by its complementary counterparts, the Trx and Grx systems.

## 2. GSH Synthesis and Denitrosylation

### 2.1. GSH Synthesis

Glutathione, or ϒ-glutamylcysteinylglycine (GSH), being the most abundant antioxidant in mammalian tissues, is imperative for several roles in the human body, which include cell proliferation, protein folding, regulation of the cell cycle and cell division, repairing DNA damage and participating in replication, redox-dependent cell signaling, reserve form of cysteine, boosting the immunogenic responses, elimination of toxicants, synthesis of eicosanoids, metal homeostasis, and storage and transportation of NO, among several others [16]. The celebration around GSH is mostly due to its connection with ROS, which not only makes it the most studied antioxidant in the field of redox biochemistry but also establishes it as a linchpin of redox-regulated cellular defenses against stress factors in a biological system, also establishing it as a very strong denitrosylating agent [17,18].

This tripeptide is a pivotal Non-Protein Thiol (NPSH) present in 5–10 mM concentrations physiologically and is synthesized in the cytosol from glutamate through a feedback mechanism [19]. Several pathways have come to light in recent days related to the biosynthesis of ϒ-glutamylcysteine from the conjugation of glutamate and cysteine; the most common being the de novo GSH synthesis through the ϒ-glutamyl cycle, involving ϒ-glutamyl transpeptidase, peptidase, and ϒ-glutamylcysteine synthetase to catalyze the biosynthesis. The same pathway can be simplified with the help of glutathione synthetases (Gsh1, Gsh2, GshF) and prolinases [20,21]. This pathway is the most crucial due to the involvement of the ϒ-glutamyl transpeptidase, the only enzyme localized on the outer plasma membrane, and is directly involved in stress-regulation and cellular GSH homeostasis, besides its role in the extracellular hydrolysis of GSH into cysteine and the intercellular resynthesis of GSH in oxidants such as human beings [22]. Another way is to conjugate cysteine with glutamate, forming ϒ-glutamylcysteine through a two-step ATP-acquiring enzymatic process involving glutamate-cysteine ligase as the catalyst for the first step [21]. The later steps of the pathway are similar for all the studied ways. ϒ-glutamylcysteine, with the help of glutathione synthetases, is reduced to form reduced glutathione (GSH), which is further oxidized (GSSG) depending on the amount of positive or negative feedback generated in the ϒ-glutamyl cycle in the presence of a particular GSH/GSSG ratio in the mammalian system [16,20].

### 2.2. S-Nitrosylation of Glutathione

**Figure 1:**
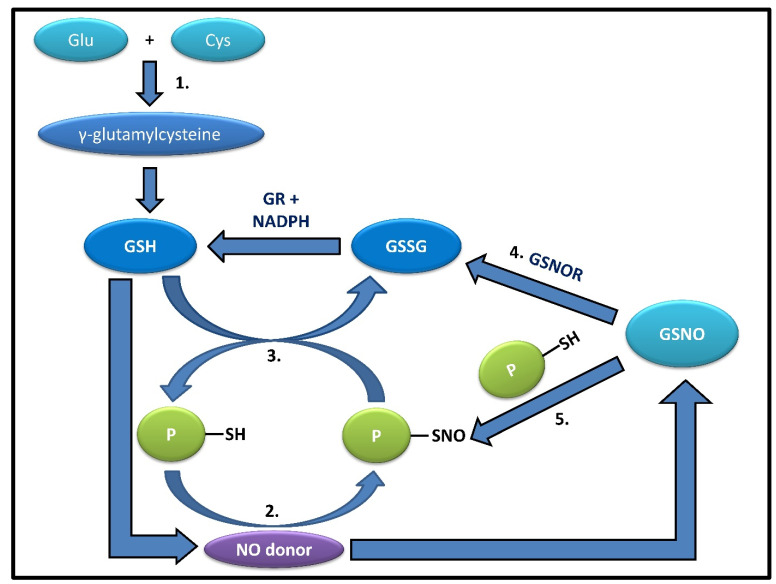
The figure shows the correlation between glutathione and its derivatives. 1.GSH can be synthesized from its precursor, γ-glutamylcysteine, derived from glutamate and cysteine.2. Most known proteins can react with NO-donors or NO_x_ species, forming labile intermediates, such as S-nitrosylated proteins (PSNOs). 3. PSNOs, except a few such as Caspase 3, can be readily denitrosylated in the presence of an abundant physiological concentration (5–10 mM) of GSH. 4. S-nitrosoglutathione reductase (GSNOR) can convert GSNO, produced by the addition of an NO donor to GSH, into oxidized glutathione (GSSG), which is recycled back to GSH by GR, utilizing NADPH as an electron source; 5. or even take part in catalyzing the trans-nitrosylation of proteins by GSNO, thus playing contrasting yet significant roles.

The addition of an NO donor to a glutathionyl radical (Figure 1) results in the formation of the most abundant low-molecular S-nitrosothiol, GSNO, which now acts as an NO reservoir and vehicle inside the cell, actively participating in cell signaling, inflammation, and neurotransmission through transnitrosation and glutathionylation. This GSNO can be decomposed to GSSG by GSNO reductase (GSNOR) [23,24]. GSNO, due to its anti-inflammatory functions, has been studied in vivo rat models for the treatment of cognitive impairment by chronic cerebral hypoperfusion, suggesting the slowing down of neurodegeneration while buying time for the patient [24]. GSNOR, on the other hand, due to its role in regulating S-nitrosothiols by protein-S-nitrosation through the breakdown of GSNO, has been identified as a potential therapeutic target for diseases ranging from tumorigenesis to neurodegeneration, thus implying an indispensable role of GSNO in the NO metabolism aiming to prevent NO-mediated toxicity in cells [25]. The S-nitrosylation of GSH thus has several benefits in the proper running of the animal systems, which again depends on a feedback mechanism of the system through proper GSH/GSSG ratio, cellular GSH level, and redox state.

If we look closely at the structure of GSH, we can gradually discover the reason for it being the most abundant antioxidant for maintaining the cellular redox status; on the one hand, the γ-peptide bond protects the biomolecule from reactive peptidases, and on the other, the -SH group at the cysteine moiety is accountable for most oxidative modifications by being a convenient electron donor to strong electrophiles. The strength of reaction of these -SH groups depends on the exposure of the cysteine moiety in its tertiary and quaternary structures, determining the strength of the oxidative balance in the cellular network. When the -SH group is exchanged by an NO donor to form GSNO, the newly formed biomolecule becomes capable of transnitrosylation, hence regulating the cellular concentration of PSNOs [26]. Now the NO donor is generated from the NOS; while the production of NO from nNOS and eNOS are comparatively low due to the involvement of calcium ions, those from iNOS are high since this NOS is mostly involved in signal transduction [27]. This reaction validates the bioavailability of NO in the mammalian system through the formation of nitrosothiols and nitrosoproteins from GSNO. This inter-balance and maintenance in the body define how complicated yet amazing the mammalian system is, which connects each biomolecule into a huge network of inter-dependency; the formation and bioavailability of NO maintained by GSH are responsible for not only the oxidative and NO stresses but also the blood pressure on a higher scale, and thus, for several cardiovascular as well as neuronal diseases [28]. Once this network is well understood and the ratios maintained, the formation of several diseases can be combated by charging the cells with an NO donor in a way for S-nitrosylation to occur and form GSNO.

### 2.3. Role of GSH in S-Denitrosylation

**Figure 2:**
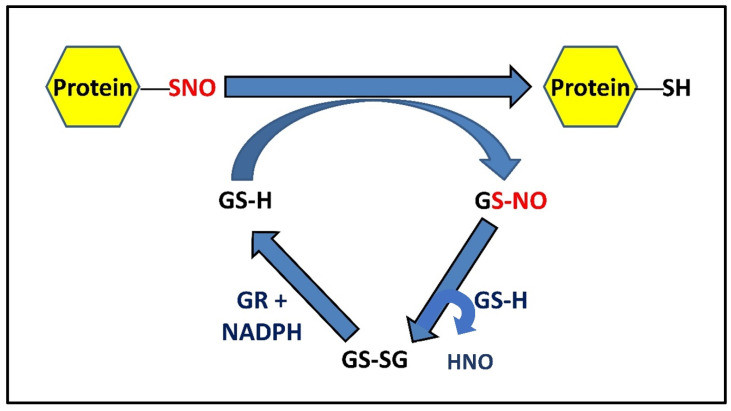
Mechanism of GSH-mediated denitrosylation of PSNOs. S-nitrosylated proteins can be denitrosylated by reduced glutathione (GSH), forming protein with reduced thiol(s) and GSNO, which is metabolized by ubiquitously expressed GSH into a stable disulfide form (GSSG) at the expense of HNO. GSSG (oxidized GSH) can be further reduced back to GSH by NADPH-dependent GR. Alternatively, GSNO can also be irreversibly metabolized by GSNOR to a complex N-hydroxysulphenamide (GSNHOH) form, utilizing reducing equivalents from NADH [3,42].

It has been well established that in active multiple sclerosis patients, there is a significant increase in the concentration of NO metabolites and low molecular mass thiols in cerebrospinal fluid. Additionally, in the white matter of these patients, there is an abundant accumulation of PSNOs with a drop in the GSH levels. Taking these facts into consideration, Romero et al., 2009 established the fact that GSH is critical for denitrosylation by conducting chase experiments in rat spinal cord slices to find relevance in the above-mentioned MS patients [29]. The spinal cord slices were incubated with GSNO, whose removal led to the speedy disappearance of PSNOs, which was further accelerated with raised levels of GSH by using the preamble analog of GSH Ethyl Ester (GSH-EE), proving that with a larger addition of GSH-EE, the rate of protein denitrosylation increases accordingly [30]. PSNO levels fall at a quicker rate in the presence of endogenous cellular GSH since the addition of GSH-EE causes an increase in NPSH concentration, which mostly consists of GSH, facilitating the increased presence of GSH, suggesting a faster rate of denitrosylation by GSH. This inverse relationship between NPSH and PSNO levels remaining after the chase keeps up with the notion that the cellular concentration of GSH is a primary determinant of nitrosothiols’ stability. Romero et al. also showed that depletion of intracellular GSH prevents protein denitrosylation [29]. When GSH was depleted with GSH depletion DEM, the steady-state level of PSNO remained stable and was higher than that in presence of GSH. Interestingly, there are reports which suggest that GSH depletion facilitates S-nitrosylation in a way where GSH will accept NO from PSNOs generating GSNO, which will, in turn, be metabolized by GSNOR [31,32,33] (Figure 1). This signifies that the depletion of GSH prevents PSNO denitrosylation and that low amounts of GSH are enough to keep protein thiols in a reduced state. Intracellular GSH was also restored by adding GSH-EE and expectedly found an increase in protein denitrosylation. Previously when GSH-EE was added, the PSNOs did not disappear completely, showing resistance to GSH, and during its replenishment, the original PSH level could not be retrieved, signifying that either the oxidized thiols are metabolically stable or inaccessible to GSH because of their location inside hydrophobic protein pockets [29]. GSH carries out the protein denitrosylation in two ways, either via transnitrosylation with PSNOs or by reaction of GSH with PSNO to form S-glutathionylated protein or PSSG (Figure 2). They stated that previously it was assumed that the increase in the rate of denitrosylation was due to the ability of GSH to intercept free NO, which is released from GSNO; rather, the actual reason is the effectiveness of GSH at reducing the generation of PSNOs [34]. All these findings suggest that decreased GSH levels lead to a decrease in the rate of S-denitrosylation by GSH, resulting in the accumulation of PSNOs in significant amounts in the white matter of MS patients helping in the aggravation of pathogenesis in active MS patients via increased nitrosative stress and protein S-nitrosylation [35]. In the study conducted by X Ren 2019, it was found that GSH denitrosylated part of HEK-PSNOs while the other part is stable to a high concentration of GSH [13]. L-Cys-SNO, GSNO, and HEK-PSNOs could be denitrosylated by Grx1 and Grx2a which have been reduced by GSH. A part of the stable PSNOs was found to become denitrosylated by Grx1 in the presence of GSH, indicating a synergistic effect on PSNOs denitrosylation and correlation between Grx and GSH in the regulation of S-denitrosylation of GSH-stable nitroso thiols [36]. A similar effect was found in Grx2a and human Grx5. Because it is the most abundant denitrosylase, it was checked whether GSH could protect other antioxidants or denitrosylases from unfitting S-nitrosylation and reported that GSH could denitrosylate inappropriately S-nitrosylated Trx1 [12]. Reports show that Trx complements the GSH-mediated denitrosylation as Trx1-SNO can be denitrosylated by reduced Trx itself. In addition to Trx1-SNO, Grx1-SNO can also be denitrosylated by GSH [12]. The study that reported GSH-mediated S-denitrosylation Grx1-SNO also reported GSH-mediated denitrosylation of R1-SNO (R1 subunit of ribonucleotide reductase [RNR]) and GSH-mediated denitrosylation of HepG2 cell-derived PSNOs by a distinctive spin trapping mechanism using *5,5 Dimethyl 1 Pyrroline 1 Oxide* (DMPO). The DMPO results showed effective denitrosylation of LMM PSNOs of HepG2 cell derived by GSH, although a population of higher molecular mass (>80 kDa) PSNOs were found to be relatively stable even in the presence of 5mM GSH [37]. Interestingly, in 2010, Benhar et al. reported that a 10 min treatment with 1mM GSH resulted in a ~75% decrease in high-MW PSNOs from Jurkat cells [38]. Although we can see GSH can denitrosylate directly, there are shreds of evidence where GSH performed denitrosylation indirectly; the most known mechanism is the formation of GSNOR and then the denitrosylation of cysteine thiols by the same. This GSNOR is active as a homodimer with the greatest activity in the liver and found in maximum content in the cytoplasm, which can also be found in the nucleus [26]. It is GSH-dependent formaldehyde dehydrogenase that can reduce GSNO [32]. It is a major regulator that can control both GSNO and PSNO levels to maintain their cellular equilibrium [3]. There was an increase in cellular levels of GSNO and PSNO when the GSNOR gene, i.e., human gene ADH5, was deleted. There is evidence that the genetic Knock Out of GSNOR in mice results in high levels of PSNO and displays multi-organ dysfunction, mortality in models of sepsis, low systemic vascular resistance, and high susceptibility to hypertension, showing that abnormal GSNOR-dependent denitrosylation is relevant to human disease [39]. As stated earlier, GSNOR does not denitrosylate proteins directly; rather, it lowers PSNO levels by pushing the equilibrium from PSNO to GSNO [40]. It is done in a way where GSH will accept NO from PSNOs to generate GSNO, which can then be metabolized by GSNOR [31]. The denitrosylation mechanism involves the reduction of PSNO to PSH and the generation of GSNO by binding one molecule of GSH [41]. This GSNO is reduced by GSNOR using NADH forming GSSG, which is also reduced by GR to replenish GSH back into the cell. The actual function of GSNOR is to metabolize or reduce the endogenously generated GSNO, whose level fluctuates markedly upon the GSH concentration depending on the nitrosative and oxidative stress inside the cell [41]. This prompt disposal of GSNO helps the equilibrium shift towards the denitrosylated state. Hence, it is evident that GSH alone cannot fully terminate SNO-signaling or protect the protein from S-nitrosylation in the absence of GSNOR [33].

### 2.4. PSNO Denitrosylation and Exceptions Amongst PSNOs

The cellular redox state is often altered by palmitoylation, henceforth affecting the functionality of cysteine thiols to maintain proper cytosolic levels of GSH required for protein denitrosylation [43,44,45]. Protein nitrosylation and denitrosylation have exerted an effect on the majority of cellular proteins. Such examples can be demonstrated in the glycolytic pathway and Kreb’s cycle where the proteins: α-ketoglutarate dehydrogenase complex (KGDHC), peroxiredoxin 6 (Prx6), aldose reductase, actin, histone H3, 5’AMP-activated protein kinase (AMPK), estrogen receptor α, heat shock protein BiP, protein disulfide isomerase (PDI), calnexin, calreticulin, and sarcoplasmic reticulum Ca^2+^-ATPase (SERCA), are also dependent on the same, for regulatory purposes. Denitrosylation of these proteins is catalyzed by certain enzymes such as GSH, glutathione peroxidase (GPx), Grx, and Trx, while the perfect ratio of GSH/GSSG is ensured by glutathione reductase (GR) and GPx [20,46].

Although cysteine residues only constitute 3% of mammalian proteins, most cysteine proteins undergo S-nitrosylation for physiological and pathophysiological roles through cellular modifications such as phosphorylation, acetylation, methylation, ubiquitination, glucuronidation, and palmitoylation [47,48,49]. The occurrence of S-glutathionylation of proteins is largely dependent on the GSH/GSSG ratio [50,51]. Since denitrosylation is catalyzed by GSH, and S-glutathionylation releases GSH, both are interdependent; results establish the direct proportionality of GSNO reduction to that of protein-SNO reduction while also suggesting the much larger redox pool of nM levels of GSNO and protein-SNO against mM level of GSH [52,53]. A broad spectrum of candidate proteins and/or enzyme substrates are central for undergoing S-(de)glutathionylation, such as GSH-dependent enzymes, including glutathione-S-transferase omega 1 (GSTO1), glutathione S-transferase P (GSTP or GSTPπ), Grx-1, GPx, peroxiredoxins, thioredoxins, membrane-associated involved in eicosanoid and glutathione metabolism (MAPEG) superfamily of enzymes, transduction and regulatory proteins (MKP-1, MAPK, PTEN, PKCα, PKCδ, MEKK1, caspase 1, caspase 8, procaspase 3, PKA, PKM, etc.), transcription factors (OxyR, Yap1p, NF-κB-p64, interferon regulator factor 3, NFκB, Nrf2, AP-1, IκB, HIF-1, STAT3), DJ-1, α-synuclein, FAS receptor, RNR, ribonuclease A, 3’-phosphoadenylylsulfate (PAPS) reductase, complex I of inner mitochondrial membrane, non-heme peroxidases, actin, histone 3 (H3), HSP70, and NaH^+^ transporter. NaK-ATPase, etc.,have been explored in the context of post-translational regulatory mechanisms that can be crucial in modulating and fine-tuning the protein/enzymatic activity, thereby restoring cellular redox balance under basal homeostatic as well as in pathophysiological conditions [54]. Similarly, about a hundred proteins are capable of S-(de)nitrosylation, another highly stringent post-translational redox modification within the intracellular milieu, where both the S-nitrosylated as well as denitrosylated proteins are responsible for cellular NO-signaling. Very few protein-SNOs are stable or are not denitrosylated in physiological GSH concentrations; these proteins include caspase-3, α-tubulin, β-tubulin, collapsin response mediator protein-2, Creatine kinase (B chain), extracellular signal-regulated kinase-2, glutathione-S-transferase (GST) pi, glyceraldehyde-3-phosphate dehydrogenase (GADPH), hemoglobin (β chain), pyruvate kinase, and Prx6 [29,55,56,57]. Amongst all these protein-SNOs, the most prominent is caspase-3, which cannot be denitrosylated by GSH or GSNOR, but exclusively by Trx; this proves the S-nitrosylation of this apoptotic protein by NO donors, but the failure of the activity regeneration in the absence of Trx, which again establishes a link between caspases and cancer and eliminates the direct role of GSH in the same, while indirectly GSH as well as reduced human Trxs have the potential to denitrosylated S-nitroso thioredoxins, inactivating caspase-3, linking GSH to the apoptotic pathways [12,58]. All these co-relations between the proteins and the antioxidant entrench a bigger picture, linking the well-established roles of GSH to tumor progression and regression while also suggesting the importance of the maintenance of the GSH/GSSG ratio.

## 3. Grx-Mediated denitrosylation and Deglutathionylation

**Figure 3:**
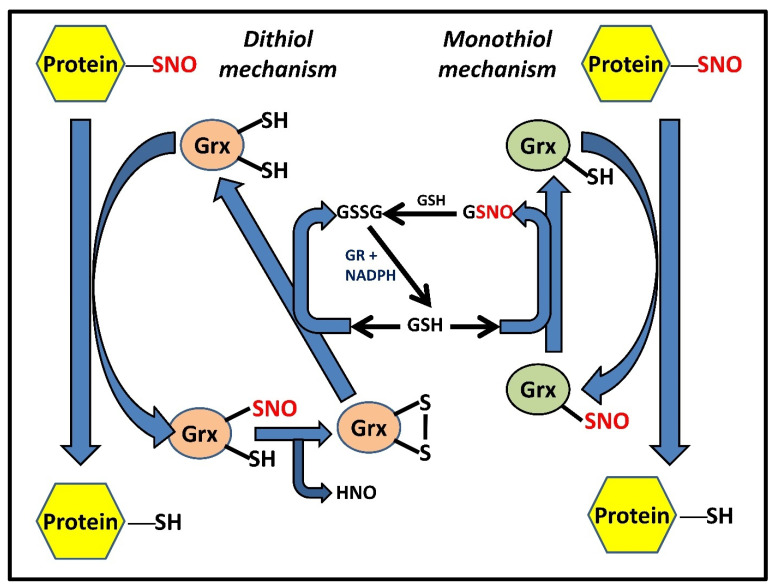
Grx-catalyzed denitrosylation of PSNOs via dithiol and monothiol mechanisms. Two distinct yet functionally similar Grx-dependent mechanisms have been proposed that can suffice for protein denitrosylation [13]; (a) the dithiol mechanism wherein Grx shuttles between three competent oxidant states, namely the reduced dithiol Grx-(SH)_2_, an intermediate Grx-(SNO)(SH), and the oxidized disulfide Grx-S_2_ form, (b) the monothiol mechanism involving Grx with only an active N-terminal cysteine residue Grx-(SH) and the Grx-SNO form. While the figure shows GSH dependency in both systems for continuous enzymatic turnover, it also highlights additional disparities between the mechanisms, such as the coupling of GSH with monothiol Grx for exhibiting denitrosylase activity, unlike its dithiol form.

In addition to the copious amounts of reduced intracellular GSH, which undoubtedly is the cell’s most effective defense against oxidative stress, the cell employs a multitude of other potent, adjunct enzymatic systems that complement the antioxidant activity of GSH and are, in turn, regulated by it. Evidence from numerous research demonstrates the synergistic as well as the additive nature of the interaction of GSH with these enzymatic systems, such as the Trx and Grx systems. The Grx family has many isoforms of Grxs, which vary in the number of cysteines in their active sites as well as where they dwell intracellularly. While Grx3 and Grx5 are mitochondrial and have only one cysteine in their active site, Grx1 and Grx2 are largely cytosolic (although Grx2a is found in the mitochondrial matrix) and marked by the presence of a dyad of redox-active thiols, instead of one. The mechanisms of denitrosylation of the dithiol and monothiol Grxs are only marginally different. In the dithiol mechanism, the two cysteines are present in a CXXC motif and are distinguished by a difference in their pKa values. This difference causes one of the cysteines to ionize at the physiological pH and causes the other to remain in the protonated form. The NO attached to substrate nitrosothiols is quickly exchanged by the ionized cysteine, thus reducing them. Subsequent formation of a redox-active disulfide bridge causes Grx to quickly transform from its intermediate nitrosylated form to its completely oxidized form (accompanied by the concomitant release of nitroxyl [HNO] radical). Two molecules of GSH are necessary for the subsequent restoration of Grx activity through the reduction of oxidized Grx. In the monothiol process, the solitary cysteine in the active site directly reduces the substrate RSNOs, which is followed by the production of Grx-SNO, which is then subsequently acted upon and reduced by a molecule of GSH, releasing GSNO in the process (Figure 3). GSNO can then either serve as an NO donor, S-nitrosylating protein thiols, or it can combine with another GSH molecule to produce GSSG, which could trigger the onset of another well-known post-translational modification called S-glutathionylation. In order to convert GSSG into two molecules of GSH, GR could act on it (in the presence of NADPH) or, in the presence of NADH, GSNO could also be effectively reduced by GSNOR or Alcohol dehydrogenase III (ADH-III). Grxs are known to denitrosylate CysNO, GSNO, and PSNOs derived from HEK cells (HEK-PSNOs), S-nitrosocaspase 3, and S-nitroso cathepsin B [29].

Grx has the ability to deglutathionylate proteins in addition to its denitrosylase function. Cysteine thiols are covalently modified with GSH to create RSSG (i.e., glutathionylated derivatives of proteins) through glutathionylation. Reduced GSH, as well as GSSG, can directly combine with RSH or RSNOs to form RSSGs. It is interesting to note that both monothiol and dithiol Grxs can reverse S-glutathionylation. Mechanisms of de-glutathionylation by dithiol or monothiol Grxs are comparable to those used for denitrosylation. According to reports, S-nitrosylation and S-glutathionylation both play significant roles in the onset and progression of neurodegenerative diseases. GAPDH is S-glutathionylated, which results in the amyloid build-up and, ultimately, Alzheimer’s disease (AD). Parkinson’s disease (PD) and mitochondrial dysfunction are brought on by decreased S-glutathionylation of ATP synthase subunits. Huntington’s disease progression, on the other hand, is influenced by S-glutathionylation of the short transient receptor potential channel 5 (TRPC5). Similarly, the development and progression of PD are also accelerated by the S-nitrosylation of proteins such as DJ-1, PINK1, and Parkin. Both protein modifications (i.e., S-nitrosylation and S-glutathionylation) have a significant influence on the onset and course of illnesses, particularly neurodegenerative disorders, as shown by instances of increased cell death in Grx defective *C. elegans* models for PD [59]. The complex and delicately balanced interplay between GSH/GSSG/GSNO and Grx is thus crucial in deciding not just the destiny of aberrantly altered proteins but that of the entire cell.

## 4. Trx Isoforms and Trx-Mediated Denitrosylation

The Trx system comprising Trx, thioredoxin reductase (TrxR), a selenoenzyme, and NADPH is a small, ubiquitous, low molecular weight (LMW) (12 kDa approx.) cellular oxidoreductase, widely distributed in all living cells with a conserved sequence homology [60,61,62]. Trx, characterized by the presence of a conserved CGPC active site motif, is present as a disulfide (Trx-S_2_) in the oxidized form and as a dithiol (Trx-(SH)_2_) in its reduced form. Trx-(SH)_2_ transfers its reducing equivalents to protein disulfides and catalyzes their reduction via a reversible dithiol–disulfide exchange reaction. Exploring its 3D crystallographic structure, diversity of its isoforms and their subcellular localization, and additional, concerted efforts over the years in altering its activity turned out to be a fruitful pursuit in revealing the multifaceted roles of the Trx system at the physiological level as well as in disease pathogenesis, in addition, to serving as an important mediator of first-line antioxidant defense and redox signaling [63]. In mammals, there are three isoforms of Trx: Trx1 (*TXN1*), primarily located in the cytoplasm and translocated to the nucleus under oxidative stress or exported out of the cell;Trx2 (*TXN2*), located in the mitochondria; and the testis-specific Trxs(Sptrx-1, Sptrx-2, and Sptrx-3) Compared to its bacterial counterparts, human cytosolic Trx1 is characterized by three additional cysteine residues, Cys^62^, Cys^69^, and Cys^73^, intriguingly involved in oxidative thiol modifications such as S-glutathionylation, S-nitrosylation, and disulfide formation, in addition to the active site Cys^32^ and Cys^35^ which highlight their crucial roles in the functional and redox regulation of Trx1 activity [64]. Apart from the active site Cys^32^–Cys^35^ disulfide formation, another intramolecular disulfide may be formed between Cys^62^ and Cys^69^ under nitro-oxidative stress, which leads to the reversible inactivation of the Trx1 activity, only to be profoundly reactivated or reduced by the Grx system [65,66]. Trx1 has the ability to regulate several transcription factors, such as NF-κB, AP-1, and p53, playing an important role in protecting the cell in oxidative stress conditions [67]. The ROS generated in the mitochondria under such oxidative stress conditions is regulated by the mitochondrial Trx (Trx2), which serves as a negative regulator of ASK1-dependent (Apoptosis Signal Regulating Kinase) apoptosis. The deficiency of Trx2 thus hinders the control over ROS production, leading to cell death in cardiomyocytes and neurodegeneration [68,69]. The mammalian Trx system also consists of a group of additional tissue- or organelle-specific members with a conserved CXXC motif, belonging to the diverse Trx family of proteins, namely, the testes/spermatid-specific SpTrx1, SpTrx2, and SpTrx3, which play a role in developing the sperm structure, cytosol-specific thioredoxin-like 1 (Txl1), microtubule-specific thioredoxin-like 2 (Txl2), plasma specific truncated Trx80 in immunomodulation, protein kinase C interacting cousin of thioredoxin (PICOT), important for T lymphocyte activation, Nrx with a selective suppressing role of Wnt-catenin signaling, Grx1, Grx2, Grx5, etc. [70,71].

**Figure 4:**
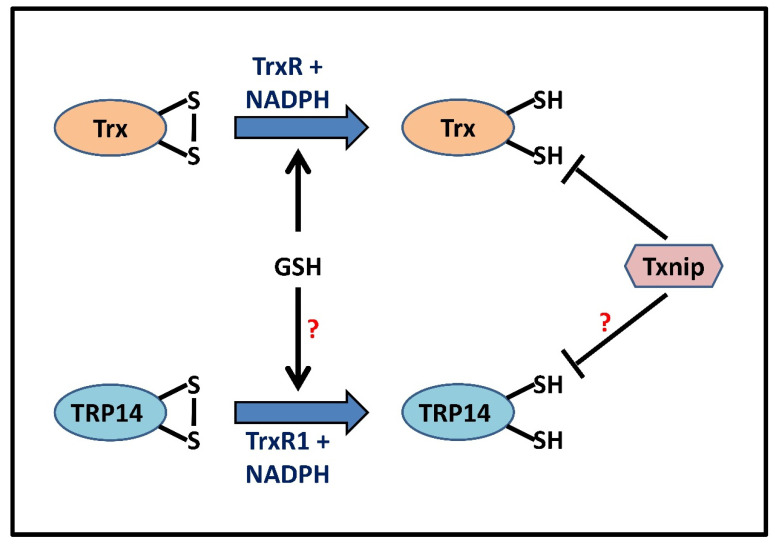
Proposed schematic representation predicting parallels between GSH and redoxin systems. Oxidized Trx (Trx-S_2_) is reduced back to its vicinal dithiol (Trx-(SH)_2_) form by TrxR, utilizing NADPH as an electron donor. TRP14, with a redox potential similar to that of Trx1, proceeds in a similar way that uses both its active site thiols in a TrxR1-dependent NADPH oxidation. GSH (5–10 mM), stimulated by the Grx/GR system, can directly reduce Trx in the absence of TrxR, thus acting as a cellular backup, whereas Txnip inhibits the reducing activity of Trx via a disulfide exchange reaction [77,78]. This figure also hypothesizes GSH-catalyzed reduction and Txnip-mediated inhibition of TRP14 activity in a manner analogous to that of Trx.

The NADPH-dependent conversion of Trx from its oxidized form into its corresponding dithiol is essentially catalyzed by a homodimeric flavoprotein TrxR (Figure 4). Mammalian TrxRs (114 kDa or more) consist of two active sites, the first one being Cys^59^-Val-Asn-Val-Gly-Cys^64^, located in the FAD domain, and the second one being a conserved active site Gly-Cys^497^-SeCys^498^-Gly, located at its flexible C-terminal tail, which along with the redox-active SeCys residue, explains their broad substrate specificity compared to their bacterial, fungal, and plant counterparts. The reaction mechanism for reducing Trx and other substrates begins with NADPH binding to the oxidized enzyme. Being a flavoprotein, TrxR utilizes its FAD domain to transfer electrons from NADPH to the first active site (CVNVGC), present as a disulfide. Reduction of the first active site results in an intermediate Cys^59^ with intact thiol and FAD bound Cys^64^, further reduced to a dithiol pair. The second half-reaction involves the transfer of electrons to the C-terminal selenylsulfide (Cys^497^–Cys^498^) in another redox-active site (GCUG) in the neighboring subunit, which in turn, is reduced to a selenol thiol pair. At physiological pH, the selenol is converted into a corresponding selenolate anion that carries out a nucleophilic attack on the disulfide of the oxidized Trx, forming an enzyme–substrate intermediate. Further, Cys^497^ attacks this bond and forms an eight-membered ring of the C-terminal by participating in a selenylsulfide bond with SeCys. In order to keep the SeCys in its anionic form, Cys^59^ will donate its electrons, thereby maintaining the enzyme in its reduced form. Thus, the dual nature of the selenocysteine moiety to act as a nucleophile and an electrophile confers a reductase activity to the enzyme towards a vast plethora of protein as well as non-protein TrxR substrates such as oxidized Trx, Grx2, PDI, and certain small molecules including selenites, dehydroascorbate, DTNB, alloxan, hydrogen peroxide, lipoic acid, cytochrome c, ubiquinone, and motexafin gadolinium [72,73,74]. In mammals, TrxR exists in three isoforms as well, the cytosolic TrxR1 (*TXNRD1*), mitochondrial TrxR2 (*TXNRD2*), and testes-specific TrxR3 or TGR (*TXNRD3*), a unique enzyme with an additional N-terminal Grx domain, mainly found in maturing sperm [74]. TrxR1 has been shown to be particularly dependent upon the selenocysteine residue and follows a fixed pathway for reducing substrates due to the restrictive movement of the C-terminal, which is controlled by the ‘guiding bar’ made up of side chains of Asn^418^, Asn^419^, and Trp^407^ [75,76]. Within our cells, certain enzymes of the same oxidoreductase family share similarities with Trx in their active site sequence or structural conformation, popularly reckoned as the Trx fold [62]. However, these proteins have specific distinguishable features which enable them to have certain functions that contribute to a nature that is different from Trxs. Such proteins are known as Trx-related proteins. TRP14 and TRP32 are the most notable and well characterized in the group of Trx-related proteins observed to date.

Trx-related protein 14 (TRP14, also called *TXNDC17/TXNL5*) is a cytosolic protein with a molecular weight of 14kDa, the oxidized form of which is ideally reduced by TrxR1 in an NADPH-dependent reaction [79]. Structurally similar to Trx1, the protein is expressed at comparatively lower levels and exhibits similar disulfide reductase activity on most substrates except for the three well-known Trx1 substrates RNR, peroxiredoxin (Prx), and methionine sulfoxide reductase (Msr). However, the active site CPDC motif slightly differs from that of Trx1 resulting in different redox potentials, which in turn accounts for their different substrate specificities [79,80]. This difference in substrate specificities is well demonstrated in the inhibition of TNF-α signaling and in the capability of TRP14 to reduce H_2_O_2_ at high concentrations [14,81]. TRP14 contains three additional cysteines (Cys^64^, Cys^69^, Cys^110^) along with the active site cysteines at positions 43 and 46, respectively. However, the non-active site cysteines do not participate in the redox reactions [79]. TRP14 has been shown to play various roles in response to changes in cellular redox state, such as inhibiting the TNF-α and NF-κB signaling pathways, reducing L-cysteine and persulfide moieties on Cys residues, exhibiting peroxidase activity, inhibiting the differentiation of osteoclasts, providing cisplatin resistance by the induction of autophagy in ovarian cancer cells, and mediating S-denitrosylation of RSNOs (L-CysSNO) and PSNOs (Caspase3-SNO, Cathepsin B-SNO, etc.), thereby warranting the capacity to modulate several cellular redox signaling pathways [14,81,82,83,84]. Interestingly, TRP14 is a good substrate only for TrxR1 and, when coupled with it, exhibits a more pronounced disulfide reductase and denitrosylase activity than Trx1; a higher catalytic efficiency (*k*cat/*Km*) for TrxR1 mediated reduction of TRP14 than Trx1 and a lower p*K_a_* value (~6.1) contribute to its better reducing ability [79]. Being able to be reduced only by TrxR1 but not supporting the classic substrates of Trx1 makes TRP14 a perfect candidate for redox signaling regulation, with unique additional yet uncharacterized functions complementing that of Trx1.

Sharing the similar active site sequence of Cys-Gly-Pro-Cys and functionality with mammalian thioredoxin, another interesting member of the Trx superfamily is the Trx-related protein 32 (TRP32, also called *TXL* or *TXNL*), a novel LMW (32 kDa) protein, ubiquitously expressed within the cytoplasm and nucleus, bearing an N-terminal Trx domain that caters to its reducing potential [85]. Despite the high similarities with Trx1, TRP32 exhibits comparable functional and structural differences, including a four-fold lesser reductase activity and higher sensitivity to oxidation. In contrast, TRP14 is reportedly resistant to inhibitory oxidation in the presence of a high concentration of oxidants, and thus, it would be interesting to speculate on its predominant roles under oxidative stress-mediated Trx1 inactivated conditions. In vertebrates, the glycine residue preceding the active site CGPC sequence in Trx is substituted by a tryptophan residue in TRP32, thereby lowering its disulfide reductase activity [86]. Trx32 is suggested to be evolutionarily conserved among multicellular eukaryotes, and the most significant uniquity to the structure of TRP32 is given by its C-terminal DUF1000 domain. This domain has been implicated in protein degradation by associating with the 26S proteasome through a thiol group in its binding site and in the reduction of the catalytic cysteine in PRL, thereby activating it for cancer metastasis [87,88,89]. In conditions of glucose deprivation as well, TRP32 overexpression is shown to assist in cellular protection, though the mechanism behind the same is yet to be elucidated [86]. Trx2 and nucleoredoxin are two recently reported yet sparsely characterized mammalian Trx-related proteins with restricted subcellular localization in mitochondria and nucleus, respectively, which might hint toward their specific compartmentalized roles within the cytoplasmic milieu [85].

Often involved in the pathophysiology of many diseases and complex disorders, including Alzheimer’s, cancer, and inflammation, the causes of which are frequently attributed to oxidative stress, there is a dysregulation observed in the levels of a certain redox-sensitive signaling protein, thioredoxin interacting protein (Txnip), that further triggers or aggravates the diseased state [90]. Txnip (also referred to as thioredoxin binding protein 2 or Vitamin-D3-upregulated protein), a member of the α-arrestin protein family, is ideally synthesized at the metabolically essential sites and was first identified as a Vitamin D3 upregulated gene (VDUP1) in HL-60 cells [91]. It inhibits the activity of reduced thioredoxin (Trx-(SH)_2_) or limits its bioavailability, the functionality attributed to the disulfide exchange reaction occurring between an intramolecular Txnip disulfide (Cys^63^℃Cys^247^) and reduced Trx, thereby potentiating its role as an oxidative stress mediator and a negative regulator of Trx. In particular, the disulfide bond is formed between Cys^247^ of Txnip and Cys^32^ of Trx [77]. Consistent with this role, Txnip overexpression extends its potency in increasing intracellular ROS levels, inhibition of growth and hypertrophy, leading to G0/G1 cell cycle arrest and apoptosis, and has been documented in type 1 and type 2 diabetes mellitus, cardiovascular disorders, cataract, and neurodegeneration, whereas loss of its expression is emphasized in tumor cells (mostly solid cancers) and metastasis [92]. Thus, this small cellular redox player (38 kDa) has garnered considerable research attention, further dissecting its roles in both redox-dependent and redox-independent conditions, such as maintaining glucose and lipid metabolism, regulating energy expenditure, cellular protection against hypoxic injury, adipogenesis, tumor-suppressive activity, transcriptional repression, inhibition of the mammalian target of rapamycin (mTOR) activity, and activation of inflammatory, metabolic and apoptotic pathways [92,93,94,95].

Trx can reduce disulfides as well as denitrosylate RSNOs/PSNOs in a likely similar biochemical mechanism, and this capability is attributed to its redox-active dithiol (Cys^32^ and Cys^35^) moiety. In order to reduce disulfides (-S-S-) or S-NOs, Cys^32^ first carries out a nucleophilic attack on the protein/substrate disulfide bond, giving rise to an intermediate wherein Trx is covalently linked with the substrate via a disulfide bridge. Cys^35^, earlier buried in Trx, now is deprotonated and carries out a second nucleophilic attack on the mixed disulfide bond, thereby forming a reduced protein thiol and oxidized thioredoxin; (Trx-S_2_) is further rapidly reduced back to its active form (Trx-(SH)_2_) by TrxR and NADPH [96] (Figure 4). The deprotonation of Cys^32^, which enables it to carry out a nucleophilic attack, can be attributed to Cys^32^ having a low pKa [97]. On the other hand, Cys^35^ has a high pKa value, and its deprotonation in *E. coli* Trx was earlier proposed to be performed by the Asp^26^ in its vicinity. However, Carvalho et al. proposed that the Cys^35^ was deprotonated by the leaving group, activating it for the second nucleophilic attack [42,98,99,100]. An alternative hypothesized model gaining impetus on Trx-based reaction mechanisms suggests that Trx can also progress in reducing S-nitrosothiols by itself being transiently S-nitrosylated, or better put, as transnitrosylated, before leading to reduced protein thiol and oxidized Trx formation, at the expense of HNO or NO [42]. Moreover, S-nitrosylated Trx can further engage in transnitrosylation by transferring its own S-NO moiety to other target proteins, such as Caspase 3. Accordingly, it remains imperative to predict the preferential reaction mechanism between Trx-catalyzed S-denitrosylation and/or trans-S-nitrosylation under basal and altered redox state within the complex cellular milieu. Consistent with the novelty of further findings, inhibition of the redox-active cysteines of mammalian Trx1, coupled with the nitrosative (PS-NO) modification of only structural Cys residues (Cys^62^, Cys^69^, and Cys^73^), mainly Cys^69^ and Cys^73^, can bring its hypothesized transnitrosylase activity into the picture [3,101]. Trx1, when S-nitrosylated at either of its vicinal dithiols (Cys^32^ and Cys^35^), is auto-denitrosylated and leads to disulfide bond formation, further compromising Trx1 disulfide reductase and denitrosylase activity [3]. Denitrosylation of multiple mammalian PSNOs by Trx, driven by substrate proteins (both LMW and HMW) and certain intracellular regulators, can exert profound effects by modulating diverse cellular processes such as redox signaling, endocytosis, inflammation, angiogenesis, and apoptosis, etc., thereby conferring cellular protection from nitrosative stress. Regulation of this activity has been seen to be different for the various isoforms, with Trx2 acting as a denitrosylase in response to Fas-signaling or NO suppressing Txnip, allowing Trx1 to act as a denitrosylase [102,103]. S-nitrosylated Caspase-3, the pivotal enzyme in apoptosis, was reportedly denitrosylated by both Trx and TRP14 systems [14,103]. This leads to the activation of the enzyme and elicits an apoptotic response from the cell. Similarly, Trx1 can denitrosylate the S-nitrosylated caspase-8, reactivating it for the extrinsic apoptotic pathway [104]. Inhibition of iNOS in cancer cells was also seen to promote the denitrosylation of S-nitrosylated Bcl-2 by Trx, subjecting the anti-apoptotic protein to undergo ubiquitination and subsequently being degraded [105]. This characteristic of Trx to denitrosylate PSNOs, though vast, is specific, and this specificity has not yet been explained well in mammalian cells. It is noteworthy that Benhar et al. and Wu et al. devised quantitative approaches for the identification of the nitrosylated substrates capable of being denitrosylated by Trx [38,106]. However, the intracellular redox status, subcellular localization of S-nitroso-Trx1/PSNOs, substrate specificity, vicinity of strong reductants such as GSH or Trx-(SH)_2_, and stability and/or half-life of Trx1-SNO bond to be able to catalyze the NO transfer, substantiated by more in vitro and in vivo studies would be crucial determinants for delineating the possibilities of both S-denitrosylation and trans-S-nitrosylation by Trxs and further studying their impact on protein activity profiles and expression levels in normal and pathophysiological conditions.

## 5. Redundancy of Redoxin Systems

Cellular existence is based on redox homeostasis. The main pillars of this balance are the two antioxidant pathways, the Trx-mediated pathway and the glutathioredoxin-mediated pathway. However, life is as intricate as it can be. These two pathways exhibit functional redundancy; in essence, the redox system in a cell that is Trx-deficient can reprogram itself to work with the Grx system and survive [107]. Ever since their discovery as an alternative hydrogen donor system for RNR with active NADPH-dependent deoxyribonucleotide synthesis in *E. coli* mutants lacking Trxs, GSH and Grx have been eventually established as putative worthy players that play synergistic roles in maintaining redox homeostasis, along with the Trx system, under normal physiological conditions. In order to explore the contribution and impact of these redoxin systems individually on the intracellular thiol and/or redox status, as well as the counteractive antioxidant defense mechanism, it was found that selective knockdown, knockout (KO), or inhibition of the components of either of the systems led to an increased, reversible susceptibility towards nitro-oxidative stress-induced cellular damage (Table 1). The findings are anticipated to advance our understanding of how trickily the target cells (diseased or abnormal state) can dodge and/or escape treatments with Trx-TrxR/ Grx-GR inhibitors by activating the other potent redox modulators, thereby maintaining redox homeostasis and ensuring cell survival. Intriguingly, with more research and further understanding of the intricacies and crosstalk between the parallel redoxin systems, the possibility of developing alternative or compensatory regulatory mechanisms in cells deprived of either of the crucial redox components was also taken into consideration. TrxR, previously being the only physiological Trx reductant, underwent aurothioglucose (ATG) induced inhibition but exhibited no change in the cell viability along with an unaltered Trx1 redox state; the findings could be extrapolated to its reduction catalyzed by physiological GSH concentrations (5–10 mM), NADPH, and GR, strongly stimulated by Grx [78]. Cumulative pieces of evidence down the line identified Trx (Trx-S_2_) as an excellent substrate for GSH and Grx and, alternatively, oxidized GSH (GSSG) as a substrate for the Trx system, thereby establishing the eminent redundancy of either of the redoxins under nitro-oxidative stress being compensated by the other system [78,108,109,110,111,112]. This ensures cell survival and progression and further highlights a crucial perspective of potentiating current conventional or novel therapeutic approaches with careful consideration of inactivating both the GSH-Grx and Trx systems to halt the cellular viability (further discussed in Section 8).

## 6. Substrate Specificity of GSH and Redoxin Systems

Extensive research in the last two decades has revealed the presence of a wide-ranging network of intracellular denitrosylases and reductases that enable the cell to combat against and survive in the presence of continual bursts of ROS/RNS, generated as a response to various extrinsic and intrinsic stimuli; some of them have been discussed above, and dihydrolipoic acid (DHLA), ADH-III, PDI, TRP-14, are their worthy companions. The existence of 5–10 mM reduced GSH, which would seem to be more than enough to vanquish the free radical miscreants, raises the question of why the cell deploys such a sizable army of denitrosylating agents. However, one must realize that although GSH is unquestionably the commander of the army, it is incomplete and, on occasion, insufficient, without its other warriors. This discussion becomes especially pertinent when viewing the intracellular redox milieu from a biochemical perspective. Reduced GSH efficiently denitrosylates small molecule nitrosothiols and a large percentage of PSNOs; however, a subset of PSNOs continues to be stable, even in the presence of cellular levels of GSH [56,57]. This suggests the presence of preferential selectivity or specificity of one substrate over the other by various denitrosylases. Proteins have complex, three-dimensional, tertiary, and quaternary structures; nitrosylation and other covalent modifications reportedly distort these tertiary structures, which as a consequence, sterically shields and thus, greatly limits the access of the nitrosylated cysteine residues to various denitrosylases. This phenomenon especially becomes relevant in some ‘hyper-nitrosylated’ proteins, having more than one nitrosylated cysteine, which differs in their reactivity and, thus, stability in the presence of GSH. Another context of great importance is the uneven abundance and the tissue-specific distribution of various proteins throughout the body. One such protein of prime importance is caspase-3; the epidermal keratinocytes, myocardiocytes, bronchial epithelium, hepatocytes, thymocytes, and renal tubule epithelium have been documented to have high amounts of caspase-3, although the majority of neurons in the brain and spinal cord have low levels of this protein [135]. Interestingly, S-nitrosocaspase-3 (both cytosolic and mitochondrial), although stable in the presence of GSH, is successfully denitrosylated by both Trx and Grx, which in turn bears great clinical impact, as the nitrosylation of caspase-3 reportedly inhibits its function as a pro-apoptotic agent, thereby engendering a tumorigenic transformation [3,12,13,52]. The existence of such specificity towards selective substrates confers differential weight and importance to certain denitrosylases in certain tissues. Experiments conducted in the rat-spinal cord failed to detect any decline in the efficiency of denitrosylation upon the chemical inhibition of TrxR; however, HepG2 cells (a widely used cellular model for hepatic cancer) devoid of TrxR exhibited a significant reduction in the denitrosylation of caspase-3 [12,29]. This further exemplifies the differential roles played by different denitrosylases under different circumstances. In addition to caspase-3, S-nitrosylated forms of several other proteins such asβ-tubulin, GAPDH, creatine kinase, and GST pi are tolerant to the high levels of reduced GSH normally present within the cells; a study conducted in 2014 provided an extensive list of putative Trx substrates, which, although they remain to be experimentally validated, notably includes all the above mentioned PSNOs [38,56].

Several studies have also reported that denitrosylases possess various overlapping substrates, in addition to specific ones. This becomes especially significant in the context of aging, which severely impairs cellular defenses against oxidative stress due to the rapid loss of its antioxidant repertoire. The ratio of reduced to oxidized GSH (GSH: GSSG) has major clinical importance in the science of aging, especially neurodegeneration. As this ratio declines with age, the complementary denitrosylating systems assume control and attempt to restore the intracellular redox balance. The importance of the redoxin systems (a collective representative of the other inter-related denitrosylases) could be reiterated by the example of enhanced cell death in Grx defective *C. elegans* models for PD (mentioned earlier in the text). Thus, it becomes evident that both the exclusive, as well as the collective roles of various denitrosylases are quintessential not only in maintaining a healthy intracellular redox balance but also in achieving complete denitrosylation of the vast repertoire of PSNOs in the highly diverse and dynamic S-nitrosoproteome. 

## 7. Drugs and Treatments: Current Potential and Challenges

The two members of the thiol-disulfide oxidoreductase superfamily, Trx and Grx, have been unequivocally recognized as critical mediators of redox homeostasis, involved in both intra- and extra-cellular redox signaling. Dysregulated redox responses have been long known to be associated with the pathogenesis of a wide plethora of diseases, including cancer, neurodegenerative diseases, diabetes, cardiovascular diseases, and autoimmune disorders. Therefore, summarizing the functional aspect of each of the components of these thiol redox systems under physiological as well as diseased states has established them as the ‘missing link’ between cellular redox regulation, antioxidant defense, and disease pathogenesis [16,136,137,138]. Tinkering with the structure and function of the redoxin domain of proteins and their selective inhibition upon a knock-out, knock-down, or gene silencing have generated many new insights into the molecular structures and biochemistry of each of the components with novel functions. Several drugs, inhibitors, mimetics, and LMW thiols targeting either of the redoxin components have been developed to harness the therapeutic benefits, which further ascertain their increasingly important functional relevance in the growing discipline of redox biology. The development and widespread clinical utility of some of the selective natural and synthetic inhibitors mentioned in (Table 2) ideally qualify them as feasible candidates for the purposes of clinical trials. Whilst studies supporting the discovery and/or designing of novel inhibitors/drugs and their utility in targeting either the Trx or the Grx system continue to build, thereby compromising the antioxidant defense machinery of the target cells, the challenge arises in situations aimed at differentially targeting redox dysregulation brought upon by specific redox signaling systems. Further adding to the complexity, downregulating or blocking either of the thiol-dependent antioxidant systems using selective inhibitors can be significantly compensated by the cells, utilizing the reductase and denitrosylase activity of the other system, thereby cleverly exploiting the ‘redundancy’ between the Trxs and glutaredoxins to its highest potential. Combinatorial therapeutic approaches to dodge severe pathologies, mainly in certain types of cancers, utilize the combinatorial effects of both Trx and Grx system-specific inhibitors (such as auranofin and buthionine-sulfoximine, respectively) or are inclined toward designing novel drugs that can synergistically arrest both the redox regulatory pathways in vivo and in vitro, thereby sensitizing the cancer cells to growth arrest and cell death [139,140,141]. Additionally, the physiologically relevant Trx and Grx (GSNOR) systems regulate a myriad of proteins and their activity via an arguably distinguished S-(de)-nitrosylation modification within the vast cellular repertoire [36,142]. Denitrosylation, catalyzed by either direct interaction with the substrate proteins or by receptor/stimulus coupling, confers protection from nitro-oxidative stress; thus, complete blocking of the denitrosylases using inhibitors, mimetic peptides, or genetic manipulation leads to an alteration of the NADPH/NADP^+^, NADH/NAD^+^, and GSH/GSSG ratios, impairing cellular ability to revert back the oxidative damages, ultimately leading to stress-induced cell death. However, considering the implications of other cellular redox enzymes such as PDI, xanthine oxidase (XO), superoxide dismutase (SOD), GPx, or other oxidases in mediating redox signals, it will be interesting to speculate if they can catalyze denitrosylation of target protein substrates to some extent, if not a worthy alternative in the absence of the ubiquitously expressed Trx and Grx system [143,144,145,146,147].

Unlike other pathophysiological conditions, the Trx system can act as a double-edged sword in cancer progression, depending upon the stage of cancer. At the onset of cancer, it exhibits direct antioxidant properties by defending against nitro-oxidative stress caused by carcinogens and xenobiotics, along with the support of other antioxidant molecules/enzymes such as GSH, Prx, and Msr, thereby preventing cell malignancy and further metastasis. However, once tumor formation has initiated, elevated levels of Trx/TrxR relative to normal cells help in coping with elevated ROS levels and facilitate tumor progression and metastasis owing to their growth-promoting, apoptosis-resisting, and angiogenesis-supporting functions, implicating themselves as crucial harbingers of chemotherapeutic resistance [169,170]. Thus, many anticancer drugs, including the widely known auranofin, have been reportedly involved in research for testing their potencies against this robust antioxidant defense system in cancer cells [171,172]. Auranofin, a linear, lipophilic, gold (I) complex bearing triethyl phosphine and thioglucose tetraacetate ligands, is a widely acclaimed cytotoxic and potent anticancer agent that targets TrxR1/TrxR2 enzymes and concomitantly influences the redox status of other downstream protein targets (PTEN, PRDX1, ASK, NF-Κb, p53, AP-1, RNR) as a single drug or in combination with other significant inhibitors, reaping huge clinical benefits in different types of cancers (myeloma, leukemia, lymphoma, breast cancer, colon, gastric, lung, and ovarian cancer, etc.) [172]. Despite the growing interest in developing novel drugs for antioxidant-based therapies, potentiating their efficacies in clinical trials remains at a standstill. Higher reactivity but a lower abundance of selenol groups compared to thiols, risks of cross-reactivity with other enzymes or cellular thiols, lack of major binding sites for inhibitors/ complexes, small surface area, and weak interactions between Trx-TrxR complex are some of the major challenges that limit the possibilities of drug designing concerning these areas [173]. Small derivatives or LMW dithiols might address some of these limitations by binding to the small interface and disrupting the enzyme-substrate association; a competitive inhibition and comparably higher enzyme specificity than Trx reasons for their inhibitory action on TrxR with high selectivity. In other clinical pathologies such as atherosclerosis, cardiovascular diseases, neurodegeneration, and inflammatory immune responses, the first line of antioxidant defense machinery, mainly the highly conserved Trx1, exerts anti-oxidative, anti-inflammatory, and atheroprotective effects. This is often compromised upon its in vivo cleavage into a truncated pro-inflammatory protein, Trx80 [71]. Significant research focused in this direction has been pivotal in exploring new horizons with the designing of small, specific, biologically active novel thiol-amides and mimetic peptides derived from the catalytic CXXC/CXC motifs, namely CB3, CB4, CB6, CB16, NAC-amides which have clearly demonstrated their roles in restoring disrupted redox state, reducing inflammation, atherosclerotic lesions, inhibition of NF-κB, JNK-p38 MAPK pathways, upregulation of GSH levels in vivo and in vitro [174,175,176]. These compounds can reverse cellular oxidative stress in a manner analogous to the Trx dithiol-disulfide exchange reaction mechanism by crossing the blood–brain barrier or permeating through the cell membrane, thus garnering further interest in developing antioxidant therapeutics based on synthetic peptides as feasible alternatives in oxidative-stress-related disorders [71,175,177,178].

## 8. Conclusions

The flexible modulation of covalently modifiable cysteine thiol(ates)s, through oxidative and nitrosative derivatization with moieties such as NO and related free radicals, encompasses a highly relevant yet largely unexplored arena of modern research, bearing major clinical significance. Within the vast cellular repertoire, the redox homeostasis is maintained by a unique S-nitrosylation/S-denitrosylation dependent redox switch, which, when dysregulated, alleviates nitro-oxidative stress that further orchestrates cellular dysfunction implicated in a wide plethora of diseases and disorders. Skepticism pertaining to the putative targets of S-nitrosylation of various proteins, and the consequent functional implications of such modifications, comprise a significant portion of the largely incomplete jigsaw of S-nitrosoproteome study. Available evidence points towards a detrimental impact of such aberrant modifications on normal protein activity, thereby directly linking them to exacerbation of the progression of multiple malignancies, metabolic disorders, and neurodegenerative diseases. Owing to the expansive distribution of proteins susceptible to S-nitrosylation all over the body, it becomes essential to take into account the tissue-specific differences in endogenous ROS/RNS production and, thereby, the local distinctions in the extent of susceptibility of various mature, differentiated cells to oxidative and nitrosative stress. A crucial factor that becomes unavoidable in this context is the uneven and tissue-specific disposition of a rather diverse assortment of denitrosylating agents. The astute exploitation of the wide plethora of denitrosylating mechanisms (discussed earlier) by the cell highlights the critical roles played by the synergistic interaction of a pair of factors, namely the ‘redundancy’ and ‘specificity’ of denitrosylases. The existence of this dual mode of action ensures that differentiated cells of various types, existing in widely different tissues, are able to put up optimal combat against the constantly fluctuating levels and types of prooxidants. As elaborated earlier, redundancy refers to the existence of two or more denitrosylating systems with overlapping substrate specificities, i.e., the presence of more than one agent capable of reversing detrimental modifications to the redox active thiols of key cellular proteins. A prime instance of such a scenario would be the presence of overlapping denitrosylation substrates of GSH, Trx, and Grx (along with related enzymes). As reiterated earlier, the presence of such cellular backups is critical for ensuring an uninterrupted and accurate service by critical proteins in the event of failure or suboptimal activity of one of these systems, owing to extrinsic homeostatic perturbations or natural processes such as aging. Examples of such cases would be the denitrosylation of RNR- large subunit by both GSH and Trx, denitrosylation of S-nitrosocaspase-3 by both Trx and Grx, etc. Similarly, ‘substrate specificity’ is a phenomenon of equal gravity; the existence of a subset of S-nitrosylated substrates specific to certain denitrosylases stems from differences in both the physicochemical properties of polypeptides and their widely distributed spatial organizations [37,179]. This inequality in the denitrosylating potentials of various denitrosylases ensures collective, efficient, and complete eradication of harmful free radicals, thereby serving to attenuate redox imbalance under conditions of stress. Stoyanovsky et al., in 2014, perspicuously demonstrated through a series of in-vitro and ex-vivo studies the concerted action of GSH and Trx, which was capable of denitrosylating the complete set of HepG2 cell-derived PSNOs, thereby re-emphasizing the collective/additive power of the multiple accessory denitrosylating systems, present in addition to 5–10 mM GSH—the master antioxidant [52]. Considerable research into gaining a deeper understanding of the complex, inter-twined properties of the intracellular denitrosylating network, using rapid, effective, and accurate proteomic and biochemical techniques, is elementary in order to enable scientists to devise efficient and prompt diagnostic tools and therapeutic molecules. Decrypting the complex overlap between denitrosylases and their multiple substrates would thereby aid in mustering missing pieces of information and provide crucial answers to open-ended concerns, such as the putative reduction of TRP14 by GSH or the possibility of Txnip mediated inhibition of TRP14 (Figure 4). Keeping in mind the novel additions to the Trx superfamily, such as TRP14, and its biochemical characterization as a comparably efficient disulfide reductase as Trx, careful consideration of exploring and expanding the substrate-specificities of GSH and Txnip on TRP14 seems like a notion possible to reconcile experimentally, thereby improving its propensity as another attractive target for drug designing.

Accurate comprehension of the actual physiochemical modeling of existing denitrosylases would also further the progress of redox science in the realm of nanomedicine—an emerging and actively cultivated arena in contemporary therapy. Upgrading the science of personalized medicine through accurate decoding of the underlying genetic and epigenetic basis of a disease would enable researchers to exploit the ‘redundant’ and ‘substrate-specific’ nature of denitrosylases and design therapies based on the tissue-specific and substrate-specific administration of cognate denitrosylating agents/antioxidants. Though the precise mechanism of this crosstalk and interplay remains elusive, their consequences are certainly noteworthy, which might further necessitate revisiting the mechanism and impact of inhibition using specific drugs or inhibitors targeting the redoxin systems to culminate their potencies in novel therapeutic strategies.

## Figures and Tables

**Table 1:** Summarization of the specificity of Grx and Trx systems in altered redox environments supported byin vivo and in vitro experimental findings.

System	Target	Genetic Manipulation Induced Functional Changes	References
Grx system	Grx1 knockout	-Lack of susceptibility of Grx1 deficient mice towards I/R heart and lungs injury in vivo -Limited role of Grx1 in hyperoxia-induced lung injury -Desensitizes Grx1 deficient MEFs towards H_2_O_2_ and diamide-induced apoptotic cell death; however, increases vulnerability to diquat and paraquat-induced cell damage	[113]
	Grx1 knockout	-Increases lens susceptibility to UVR-B-induced oxidative stress -Highlights in vivo impact of Grx1 in protection from UVR-induced subcapsular and cortical cataract	[114]
	Grx2 knockout	-Exhibits reduced tolerance to oxidative stress -Suppression of mitochondrial protein complex I and IV activity upon S-glutathionylation and enhanced ATP loss -Accelerates early onset and progression of cataract -Impairs cell viability and membrane integrity under H_2_O_2_-induced stress in primary LECs	[115,116,117]
	Grx1 overexpression and knockdown	-Offers better resistance to H_2_O_2_-induced Akt glutathionylation and Caspase-3 mediated cell viability loss -Activation of Akt upon phosphorylation followed by decreased Bax and increased Bcl-2 expression levels -Sensitization of Grx-1 KO ARPE-19 cells to apoptosis	[118]
	Grx1 knockdown and overexpression	-Decrease in GSH/GSSG ratio and cellular GSH levels -Increased ROS accumulation and glutathionylation in DJ-1 and HSP60 -Inactivation of DNA replication and damage repair pathways -Induces cell cycle arrest via p53/p21/p16 signaling axis activation in 293T and U87 cells -Decrease in cellular ROS levels upon overexpression	[119]
	Grx1 knockout	-Induces cellular copper retention and reduction in copper-induced oxidative stress tolerance -Highlights role of Grx1 in ATP7A-mediated copper export, maintenance of neuronal copper homeostasis in AD, PD, and ALS -Offers protection from copper-mediated oxidative injury	[120]
	Downregulation of Grx1 and Grx1 overexpression	-Mitochondrial dysfunction and loss of MMP and mitochondrial membrane integrity upon oxidative insults in neuroblastoma cell lines -Oxidative modifications in redox-sensitive VDAC and increase in ROS generation -Desensitizes cells against L-BOAA cytotoxicity upon Grx1 upregulation	[121]
	Depletion of GSH and Gpx2 levels, Knockdown of Gpx2 or GSTA2 combined with GSH depletion	-Impairs iPSC-specific resistance to mt and nuclear DNA damage upon H_2_O_2_ exposure -Strong increase in DNA damage (GSH-depleted fibroblasts) and considerable increase in ROS levels (GSH-depleted fibroblasts and iPSC)	[122]
	Overexpressed mtGrx2 and truncated cytosolic Grx2 (tGrx2)	-Lower susceptibility to apoptosis in both forms -Lesser susceptibility of mtGrx2 than tGrx2 towards apoptosis -Inhibition of cytochrome *c* release, caspase3 activation, and cardiolipin loss	[123]
Trx and TrxR	TrxR2 (*Txnrd2*) knockout	-Inactivation of Hif-1α signaling, Hif-1α degradation, decreased VEGF levels, increased JNK activation -Delays angiogenic switch, reduces tumor growth, and impairs angiogenesis -Displays higher ROS levels than wt MEFs despite strong, compensatory upregulation of Grx2	[124]
	Heart-specific TrxR2 (*Txnrd2*) knockout	-Deregulated autophagic activity in cardiomyocytes -Decreases O_2_ consumption, elevates ROS production, loss of morphological and functional integrity of KO mitochondria -Heightened catalase, HSP25, HSP60, and GSH levels in KO myocardium	[125]
	TrxR1 knockdown	-Increase in some selenocysteine prodrugs-mediated cytotoxicity in vivo -Increase in ROS generation, selenocompounds-induced mitochondrial membrane depolarization, DNA strand breaks, and AIF-induced cell death in human lung cancer cells	[126]
	Heart-specific TrxR1 and TrxR2 knockout, Trx2 overexpression	-Aggravated systolic dysfunction and myocardial cell death -Attenuates oxidative stress, mitochondrial impairment, loss of membrane integrity, and larger infarct size in clinical testings -Exhibits more rate-limiting relevance of TrxR2 as compared to Trx2	[127]
	TrxR1 knockdown	-Reversal of numerous malignant properties, including tumorigenicity in malignant mouse cell line and mouse model -Defective S-phase cell progression in serum-deficient medium -Decrease in DNA polymerase α expression leading to inhibition of DNA replication	[128]
	TrxR1 knockdown	-Alters relative levels of reduced (decrease) and oxidized (40% increase) Trx1 in KO cells upon H_2_O_2_ exposure -Unaffected redox status of Trx1 under normal KO cells	[129]
	TrxR1 inactivation	-Leads to early embryonic lethality -Severe growth retardation (homozygous mutant)in vivo and in vitro, unaffected embryonic fibroblasts (ex vivo) -Reveals higher significance of TrxR2 in cardiogenesis and TrxR1 in other developing tissues	[130]
	Trx-deficient mice	-Increases endogenous Trx oxidation, p53 and Gadd45α expression, and synthesis of proinflammatory cytokines in normal O_2_ levels -Exhibits significant mortality in hyperoxic conditions -Decrease in aconitase and NADPH activity, impairs mitochondrial energy metabolism	[131]
	Cytosolic TrxR1 and mitochondrial TrxR2 overexpression	-Downregulates GPx expression and activity levels upon elevated TrxR1 levels -Induces novel expression of epithelial markers in HEK-293 cells for cellular differentiation	[132]
	ß-cell-specific TrxR1 knockout	-Increases sensitivity to oxidative damage -Lowers glucose-stimulated or membrane depolarization stimulated insulin secretion -Upregulates Nrf2-related antioxidant genes, altered expression of heme and GSH-related genes -Downregulates β-cell function and identity regulating factors	[133]
	Trx2, TrxR2, Trx1, and TrxR1 disruption and deletion	-Sensitizes cells to elevated levels of ROS in *nuo-6* and *isp-1* mutants -Significant increase in ROS levels upon loss of TrxR2 and Trx1 -Significant shortening of lifespan, decreased heat stress resistance, decreased resistance to osmotic stress and bacterial pathogens	[134]

**Table 2:** This table highlights some of the well-established synthetic as well as natural inhibitors and further analyses their inhibitory potency based on their selective inhibition of the major redoxin systems (Grx-, Trx- system, or both).

Class of Inhibitors	Name of Compound/Complex/Drug Candidate	Outcome of Inhibition and Potential Consequences	References
Grx, Grx-system inhibitors	BCNU	-Dramatically reduced Grx activity -Inhibition of flow-induced Akt and eNOS phosphorylation (activation) in endothelial cells -Inhibition of atheroprotective effect of Akt-eNOS-NO signaling pathway	[148]
	2-AAPA	-Irreversible inactivation of intracellular GR activity in a time- and concentration-dependent manner -Induces thiol oxidative stress -Reduction in enzyme activity, i.e., minimal inhibition against GPx and GST -Exhibits potential anticancer and antimalarial activity	[149,150]
	Cadmium	-Inhibition of thioltransferase (GSH-dependent dethiolase) activity in a dose-dependent manner -Inhibition of cellular GR activity -Accumulation of PSSG substrates in mouse neuronal (HT4) cells -Mediates apoptosis in lysates of H9 and Jurkat cells -Inhibition of Grx1 and Grx2	[151,152]
	Levodopa	-Inactivation of Grx activity in a time- and dose-dependent manner by dopaquinone adduct formation -Decreases TR and Trx content -Impairs the overall thiol homeostasis -Ensures apoptotic cell death in dopaminergic neurons in PD	[153]
	Sporidesmin	-Selective inactivation of Grx1 activity in a concentration-, time- and oxygen-dependent manner in the absence of GSH -Exhibits cytotoxic effects upon thiol modifications in selective target proteins	[154]
	Methylmercury	-Induces oxidative stress by disrupting GSH homeostasis in astrocytoma cells -Significant decrease in GSH/GSSG −50% decrease in Grx1 activity	[155]
Trx, TrxR inhibitors	Metal complexes or compounds containing Sn(IV), Ru(II, III, IV), Rh(I), Ag(I), Cu(I), Pt(I), Au(I)	-Selectively inhibits TrxR activity (TrxR1 and TrxR2) -Triggers mitochondrial dysfunction, DNA damage, cell cycle arrest, and apoptosis in cancer cell lines -Induces paraptosis (Cu complexes) -Time- and dose-dependent inhibition of Trx, TrxR, NADPH (Cisplatin (CDDP) -Triggers intracellular covalent complex formation of TrxR1 with Trx1 or TRP14, cytotoxicity, and anticancer efficacy	[156,157,158,159,160]
	TXNIP	-Negative regulator of Trx1 activity -Increases mitochondrial ROS accumulation -Activation of NLRP3 inflammasome -Binding of Txnip with Trx2 reduces Trx2-Ask1 interaction, further activating Ask1 for apoptosis	[136]
	Dichalcogenides and sulfur/selenium-containing compounds	-Interaction with TrxR enzymes, as inhibitors or substrates -Irreversibly alkylates Trx1 (PX-12) -Inhibits proliferation of cancer cells and reduces tumor size (Ethaselen) -Promotes oxidative stress-induced apoptosis of cancer cells (Chaetocin, gliotoxin, etc.)	[161]
	Polyphenolic compounds (curcumin, quercetin, myricetin, flavonoids, etc.)	-Induces alkylation of both cysteine and selenocysteine -Strong inhibition of mammalian TrxR activity -Significantly increases ROS production and induces oxidative stress-mediated cancer cell death	[162,163]
	Michael acceptors	-Inhibition of TrxR by covalent interaction with catalytic selenocysteine residue in enzyme’s active site -Exhibits pronounced anticancer efficacy	[160]
Miscellaneous	Coordination Sb(III) and Au(III) complexes)	-Strong inhibition of TrxR at sub-micromolar concentration -Inhibits GR at higher concentrations -Pronounced cytotoxic effect against MCF-7 and HT-29 cells	[164]
	GS-Pt chelate complex (purified glutathione adduct of cisplatin)	-Potentially inhibits Grx (human and bacterial) as well as Trx (mammalian) system	[165]
	Dp44mT and Bp44mT (Thiosemicarbazone iron chelators)	-Potent iron chelation efficacy -Inhibitory effect on RNR by altering thiol redoxin systems -Relative decrease in cellular GSH, TrxR activity, and Trx oxidation -Significant reduction of Grx activity -Exhibits potent and selective antitumor activity	[166]
	APR-246 (mutant p53-targeting compound)- MQ (Michael acceptor)	-Loss of free thiols due to covalent binding of MQ with thiols -Potentially inhibits Trx1 and Grx1, and RNR in vitro and in living cells -Exhibits GSH-dependent inhibitory efficiency -Triggers apoptosis-mediated tumor cell death due to mutant p53 reactivation	[167]
	LCS3	-Selective inhibition of lung cancer cells -Induces ROS and activates Nrf2 pathway -Mediates synergistic inhibition of GSH/Trx pathways	[168]

## Abbreviations

NO	nitric oxide
cGMP	cyclic GMP/Guanosine 3′,5′-cyclic monophosphate
NOSs	Nitric Oxide Synthases
nNOS	neuronal nitric oxide synthase
iNOS	inducible nitric oxide synthase
eNOS	endothelial nitric oxide synthase
ROS	reactive oxygen species
RNS	reactive nitrogen species
NADPH	nicotinamide adenine dinucleotide phosphate
NOX	NADPH Oxidase
PSNOs	S-nitrosoproteins
GSNO	S-nitrosoglutathione
PTEN	phosphatase and tensin homolog
FTD	frontal temporal dementia
ALS	amyotrophic lateral sclerosis
GSH	glutathion/ϒ-glutamylcysteinylglycine
Trx	thioredoxin
Grx	glutaredoxin
GSSG	oxidized glutathione
GSNOR	GSNO reductase
KGDHC	α-ketoglutarate dehydrogenase complex
Prx6	peroxiredoxin 6
AMPK	5′AMP-activated protein kinase
SERCA	sarcoplasmic reticulum Ca^2+^-ATPase
PDI	protein disulfide isomerase
GPx	glutathione peroxidase
GSH-EE	GSH Ethyl Ester
NPSH	Non-Protein Thiol
HNO	nitroxyl
GR	glutathione reductase
ADH-III	alcohol dehydrogenase III
GAPDH	glyceraldehyde-3-phosphate dehydrogenase
TrxR	thioredoxin reductase
ATG	aurothioglucose
DHLA	dihydrolipoic acid
PDI	protein disulfide isomerase
TRP14	thioredoxin-related protein 14
LMW	low molecular weight
HMW	high molecular weight
XO	xanthine oxidase
SOD	superoxide dismutase
Msr	methionine sulfoxide reductase
I/R	ischemia/reperfusion
MEF	mouse embryonic fibroblast
LEC	lens epithelial cells
ARPE-19	human retinal pigment epithelial cells
KO	knockout
AD	Alzheimer’s disease
PD	Parkinson’s disease
MMP	mitochondrial membrane potential
iPSC	induced pluripotent stem cells
Hif-1α	hypoxia-inducible factor-1α
JNK	c-Jun NH2-terminal kinase
Nrf2	nuclear factor erythroid 2-related factor 2
HSP	heat shock protein
BCNU	1:3-bis[2-chloroethyl]-1-nitrosourea
2-AAPA	2-acetylamino-3-[4-(2-acetylamino-2-carboxyethylsulfanylthiocarbonylamino)phenyl-thiocarbamoyl sulfanyl propionic acid
Akt	protein kinase B
VEGF	vascular endothelial growth factor
GST	glutathione-S-transferase
PSSG	glutathionylated protein
TXNIP	thioredoxin interacting protein, or thioredoxin binding protein-2, TBP-2, or Vit D3 upregulated protein
ASK1	apoptosis signal-regulating kinase 1
NHC complex	*N*-heterocyclic carbene complexes
RNR	ribonucleotide reductase
MQ	methylene quinuclidine
